# Gout as a Risk Factor for Dry Eye Disease: A Population-Based Cohort Study

**DOI:** 10.3390/jcm8010062

**Published:** 2019-01-09

**Authors:** Chia-Yi Lee, Hung-Chi Chen, Chi-Chin Sun, Hung-Yu Lin, Ko-Hsiu Lu, Jing-Yang Huang, Chao-Bin Yeh, Shun-Fa Yang

**Affiliations:** 1 Department of Ophthalmology, Show Chwan Memorial Hospital, Changhua 500, Taiwan; ao6u.3msn@hotmail.com (C.-Y.L.); anthonyhungyulin@hotmail.com (H.-Y.L.); 2 Department of Optometry, College of Medicine and Life Science, Chung Hwa University of Medical Technology, Tainan 717, Taiwan; 3 Department of Ophthalmology, Chang Gung Memorial Hospital, Linkou 333, Taiwan; mr3756@cgmh.org.tw; 4 Department of Medicine, Chang Gung University College of Medicine, Taoyuan 333, Taiwan; 5 Center for Tissue Engineering, Chang Gung Memorial Hospital, Linkou 333, Taiwan; 6 Department of Ophthalmology, Chang Gung Memorial Hospital, Keelung 204, Taiwan; arvin.sun@msa.hinet.net; 7 Department of Chinese Medicine, Chang Gung University, Taoyuan 333, Taiwan; 8 Institute of Medicine, Chung Shan Medical University, Taichung 402, Taiwan; 9 College of Health, Chung Chou University of Science and Technology, Changhua 500, Taiwan; 10 Department of Optometry, Yuanpei University of Medical Technology, Hsinchu 300, Taiwan; 11 School of Medicine, Chung Shan Medical University, Taichung 402, Taiwan; cshy307@csh.org.tw (K.-H.L.); sky5ff@gmail.com (C.-B.Y.); 12 Department of Orthopedics, Chung Shan Medical University Hospital, Taichung 402, Taiwan; 13 Department of Medical Research, Chung Shan Medical University Hospital, Taichung 402, Taiwan; wchinyang@gmail.com; 14 Department of Emergency Medicine, Chung Shan Medical University Hospital, Taichung 402, Taiwan

**Keywords:** gout, dry eye disease, inflammatory, population-based

## Abstract

This study evaluated the effect of gout on the risk of dry eye disease (DED) by using the National Health Insurance Research Database (NHIRD). Data for 30,192 gout patients (21,081 men and 9111 women) and 30,192 non-gout patients (21,005 men and 9187 women) were analyzed. Approximately 1 million patients were randomly sampled from the NHIRD registry. After applying exclusion criteria, patients diagnosed with gout were enrolled in the study group. Thereafter, each individual in the study group underwent the matching process via the propensity score with another non-gout individual, which constituted the control group. The main outcome was defined as the development of DED in accordance with the corresponding International Classification of Diseases, Ninth Revision. In addition to DED, other risk factors including age, sex, and urbanization, and several co-morbidities were included in the multivariate model. The incidence of DED with the adjusted hazard ratio (aHR) and cumulative probability were evaluated in the gout and non-gout patients. A total of 2913 DED events were observed in the study group, whereas 2631 DED events were observed in the control group. A higher incidence rate ratio was found in the study group after adjustment (aHR: 1.065). Moreover, the cumulative probability indicated a significantly increased risk of DED in the study group (*p* = 0.001). The other potential risk factors of DED according to the multivariate analysis include older age, female gender, higher degree of urbanization, keratopathy, age-related macular degeneration, glaucoma, cataract, ischemic heart disease, hyperlipidemia, peripheral vascular disease, chronic pulmonary disease, rheumatic disease, peptic ulcer disease, liver disease, and malignancy. In conclusion, gout increased the risk of DED after adjustment, and the risk is positively correlated to a longer disease period.

## 1. Introduction

The incidence of dry eye disease (DED), an ocular surface disorder characterized by dryness or a gritty sensation and even blurred vision or ocular pain [1,2], is increasing with a predominance of old age and female sex [2,3]. The pathophysiology of DED involves excess evaporation, aqueous deficiency, and ocular surface damage, which result in an unstable tear film [4]. Recently, the activation of various inflammatory processes, mainly the adaptive T cell-mediated response, has been proven to produce the chronic inflammatory status and tear film instability in DED, and create a vicious cycle if not retarded [4]. Common treatment strategies for DED include artificial tear lubrication, polyunsaturated fatty acid supplementation, and meibomian gland heating [5,6]. In addition, immunosuppressants, such as cyclosporine, have been used in patients with DED for anti-inflammatory effects [6].

Various autoimmune and inflammatory diseases have been reported to have a strong correlation with the development of episcleritis, uveitis, and DED [7]. Sjögren syndrome leads to a decline in the lacrimal gland tissue and results in inadequate tear secretion and subsequent DED [7]. Moreover, other inflammatory diseases, such as Stevens–Johnson syndrome, rheumatoid arthritis, and graft-versus-host disease increase the severity of DED with a higher ratio of inflammatory signs [8,9]. Because DED and systemic inflammatory disorders share a similar pathophysiology in immunoreaction [4,7], the increased systemic inflammatory status may increase the risk of DED.

Gout is a chronic disease characterized by hyperuricemia, urate crystal deposition, and inflammatory arthritis resulting from inflammation [10,11]. The association between gout and ocular disorders has been demonstrated in previous reports of crystalline maculopathy and keratitis [12,13]. However, studies have seldom investigated the relationship between gout and DED, with an inconclusive association between these two disorders [14]. Although some studies based on regional populations have been conducted to survey the correlation between gout and DED with conflicting results, the exact time sequence between gout and DED remained only partially elucidated [15,16,17,18,19]. Because gout and DED share a similar etiology of the T-cell-mediated pathway [4,11] and affect a majority of the population [3,4,10,20,21], a large-scale study must be conducted to evaluate the potential association between these two diseases.

The aim of the current study was to investigate the effect of gout on the risk of DED by using the National Health Insurance Research Database (NHIRD) in Taiwan. Other co-morbidities that may be correlated to DED were also analyzed in the multivariate model.

## 2. Experimental Section

### 2.1. Data Source

This retrospective population-based cohort study was approved by the National Health Insurance Administration and the Institutional Review Board of Chung Shan Medical University (Registration Number: CSMUH CS17075). Provided by the Taiwan National Health Research Institutes, the NHIRD contains data of insurance claims from more than 99% of Taiwan’s population. The claims data were obtained from the 2010 Longitudinal Health Insurance Database (LHID2010) in the current study. The LHID2010 contains data on 1 million patients randomly sampled from the NHIRD registry for the year 2010. The LHID2010 data were linked from 1 January 2009, to 31 December 2012, and the International Classification of Diseases, Ninth Revision (ICD-9) was used for disease diagnosis. Details on the medications prescribed for the patients and the demographics, socioeconomic status, and residence of the patients are also available in the NHIRD.

### 2.2. Patient Selection

Patients were defined as having gout if their medical records indicated a history of gout (ICD-9 code: 274.x). To avoid misdiagnosis of gout by recruiting patients with similar morbidities, including rheumatoid arthritis or psoriatic arthritis, only patients who underwent laboratory tests, including blood test (exam codes: 08011C, 08013C, 08016C, 08026C, 08036B, and 08126B), biochemistry profile (exam codes: 08005C, 09002C, 09013C, 09015C, 09025C, 09026C), and uric acid examination (exam code: 09013C), before the diagnosis of gout were enrolled. The index date was set as the date on which the first diagnosis of gout was received. To accurately elucidate the association between gout and DED, the following exclusion criteria were applied to exclude certain impaired ocular conditions: (1) receipt of a diagnosis of legal blindness (ICD-9 code: 369.4); (2) receipt of corneal transplantation (ICD-9 codes: 11.6x, V42.5, 996.51); (3) receipt of any type of eyeball removal surgery (ICD-9 codes: 16.5x); and (4) receipt of a diagnosis of DED (ICD-9 codes in the next section) before the index date. In addition, each individual in the study group was propensity score-matched with a non-gout individual, as discussed in the following sections, which constituted the control group. Patients with gout who could not be matched with non-gout patients due to extreme numbers of co-morbidities were excluded.

### 2.3. Main Outcome Measurement

In the current study, DED was diagnosed based on ICD-9-CM codes 370.33, 370.34, 372.53, 375.15, and 710.2. In practice, ICD-9 codes for “unspecific corneal disorder” and “unspecific keratitis” may also be considered for some forms of DED, but these codes were eliminated to prevent overestimation and confusion. Furthermore, only patients who received the abovementioned diagnostic codes by an ophthalmologist (department code: 10) were considered as having achieved an outcome and were included in the study.

### 2.4. Demographic Variables and Co-Morbidities

To standardize the health condition of participants, we also considered the effects of demographic conditions (i.e., age, sex, and income level) and the following systemic co-morbidities, according to our Modified Deyo–Charlson co-morbidity index in the analysis model: hypertension (ICD-9-CM codes 401–405), diabetes mellitus (DM) (ICD-9-CM codes 250.x, 277.7), ischemic heart diseases (ICD-9-CM codes 410.x, 412.x 414.0, 414.0x, 414.2, 414.3, 414.4, 414.8, and 414.9), hyperlipidemia (ICD-9-CM codes 272.0, 272.1, 272.2, 272.4, and 272.9), congestive heart failure (ICD-9-CM codes 398.91, 402.01, 402.11, 402.91, 404.01, 404.03, 404.11, 404.13, 404.91, 404.93, 425.4–425.9, 428.x), peripheral vascular disease (ICD-9-CM codes 093.0, 437.3, 440.x, 441.x, 443.1–443.9, 47.1, 557.1, 557.9, V43.4), cerebrovascular disease (ICD-9-CM codes 362.34, 430.x–438.x), dementia (ICD-9-CM codes 290.x, 294.1, 331.2), chronic pulmonary disease including asthma (ICD-9-CM codes 416.8, 416.9, 490.x–505.x, 506.4, 508.1, 508.8), rheumatic disease (ICD-9-CM codes 446.5, 710.0–710.4, 714.0–714.2, 714.8, 725.x), peptic ulcer disease (ICD-9-CM codes 531.x–534.x), liver disease (ICD-9-CM codes 070.22, 070.23, 070.32, 070.33, 070.44, 070.54, 070.6, 070.9, 456.0–456.2, 570.x, 571.x, 572.2–572.8, 573.3, 573.4, 573.8, 573.9, V42.7), hemiplegia or paraplegia (ICD-9-CM codes 334.1, 342.x, 343.x, 344.0–344.6, 344.9), renal disease (ICD-9-CM codes 403.01, 403.11, 403.91, 404.02, 404.03, 404.12, 404.13, 404.92, 404.93, 582.x, 583.0–583.7, 585.x, 586.x, 588.0, V42.0, V45.1, V56.x), and malignancy, including lymphoma and leukemia, but excluding malignant neoplasm of the skin (ICD-9-CM codes 140.x–172.x, 174.x–195.8, 200.x–208.x, 238.6). To further standardize the ocular condition, keratopathy (ICD-9-CM codes 370.0x, 370.2x, 370.3x, 370.4x, 370.5x, 370.6x, 370.8, 370.9, 371.0x, 371.21–371.23), cataract (ICD-9-CM codes 366.10–366.19, 366.8, 366.9 and surgery code: 86008C), age-related macular degeneration (AMD, ICD-9-CM codes 362.50, 362.51, 362.52), glaucoma (ICD-9-CM codes 365.1x, 365.2x, 365.7x, 365.9), and uveitis (ICD-9-CM codes 363.0x, 363.1x, 363.2x 364.0x, 364.1x, 364.2x and 364.3) were also considered in the multivariate model. We longitudinally traced the data from the index date until the date of DED diagnosis, withdrawal from the National Health Insurance program, or 31 December 2013.

### 2.5. Statistical Analysis

SAS version 9.4 (SAS Institute Inc., Cary, NC, USA) was employed for all the analyses. First, propensity score matching was used to control the potential confounders for each individual. The purpose of propensity score matching is to simplify the matching process while multiple confounders need to be considered and balanced, in which all the potential confounders or risk factors will be condensed into a single score [22,23]. For further details about propensity score matching, the Greedy algorithm was employed to balance the characteristics between the gout and control group with a 1:1 ratio of matching in the current study. The considered matching variables enrolled in the propensity score matching in the current study included birth year, sex, urbanization, and co-morbidities (and involved the systemic and ocular diseases mentioned in the “Demographic Variables and Co-Morbidities” section). The paired gout and control individuals were randomly matched when the difference in propensity score calculated through logistic regression was less than 0.01 between an individual with gout and one without. To yield an index of matching, we provided the absolute standardized difference (ASD), which implies the standardization [24], and an ASD less than 0.1 would be regarded as the balance in the baseline covariate. For those confounders with an ASD larger than 0.1, the regression model was applied to adjust the residual confounders, which are discussed in the following section. After propensity score matching, the chi-squared test was employed to assess the differences in the demographic data (age, sex, and income level) between the study and control groups. Then, the incidence rate ratio (IRR) and corresponding 95% confidence intervals (CIs) were calculated using Poisson regression. Cox proportional hazards regression was adopted to compute adjusted hazard ratios (aHR) by incorporating the aforementioned demographic data, prominent ocular diseases, and systemic co-morbidities in the multivariate model. The crude and aHR of all demographic data, prominent ocular diseases, and systemic co-morbidities were also analyzed, except the income level because there were too few individuals with a low income level to conduct the analysis. We plotted Kaplan–Meier curves to indicate the cumulative incidence proportion of DED between the study and control groups, with an interval of 168 months after gout diagnosis, and used the log rank test to determine the significant difference between the survival curves. Also, we divided the whole follow-up interval into three different periods, and the aHR between the study and control groups in a different time period was analyzed by landmark analysis. Because most patients in the NHIRD are Han Taiwanese, race was not considered as a covariate. Statistical significance was set at *p* < 0.05.

## 3. Results

**Figure 1:**
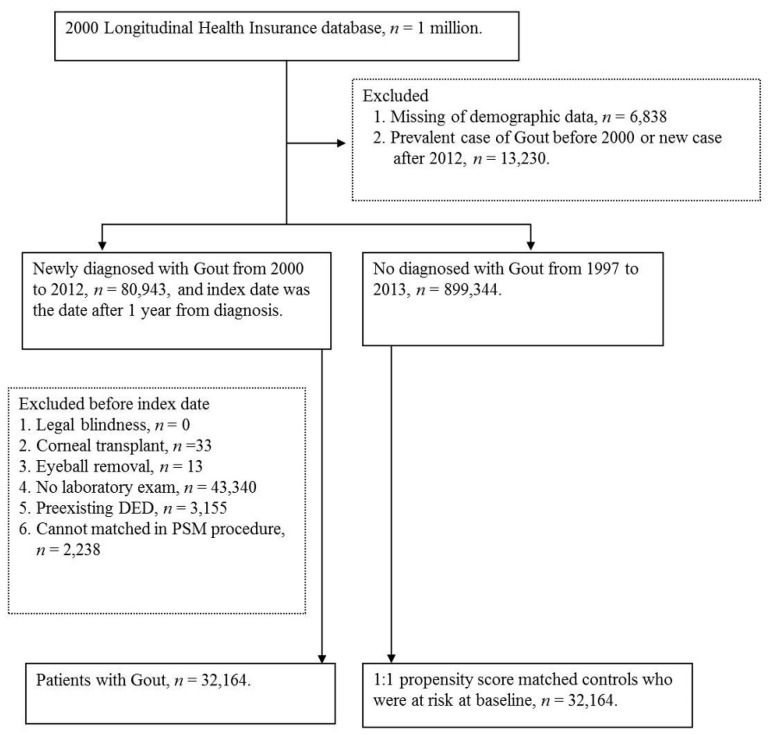
Flowchart of patient selection.

After the selection and exclusion (Figure 1), a total of 32,164 and 32,164 individuals were included in the study group and control group, respectively. The mean age was 54.86 (standard deviation (SD) = 17.02) in patients with gout, which was lower than the control group (Mean = 56.80, SD = 16.76), and the control group included more patients aged older than 60 years than did the study group (47.58% versus 42.14%). The age distribution were also significantly older in the control group (*p* < 0.0001). On the other hand, the sex and urbanization distributions showed no difference. The ratios of systemic co-morbidities and ocular disorders, with the ASD, are presented in Table 1.

A total of 2913 DED events occurred in the study group, whereas 2631 DED events were reported in the control group. The IRR (105.28, CI: 101.53–109.17) and crude HR (1.093, CI: 1.036–1.152) were significantly higher in the study group, and this group also had a significant aHR (1.065, CI: 1.009–1.126) (Table 2) after adjustment for the potential confounding from the demographic variables and co-morbidities (including glaucoma, cataract, uveitis, hypertension, DM, ischemic heart disease hyperlipidemia, congestive heart failure, peripheral vascular disease, dementia, chronic pulmonary disease, rheumatic disease, peptic ulcer disease, liver disease, hemiplegia or paraplegia, renal disease, and malignancy). The other potential risk factors of DED according to the multivariate analysis include older age, female gender, higher degree of urbanization, keratopathy, AMD, glaucoma, cataract, ischemic heart disease, hyperlipidemia, peripheral vascular disease, chronic pulmonary disease, rheumatic disease, peptic ulcer disease, liver disease, and malignancy; the crude HR, aHR, and CI of these risk factors are shown in Table 3.

**Figure 2:**
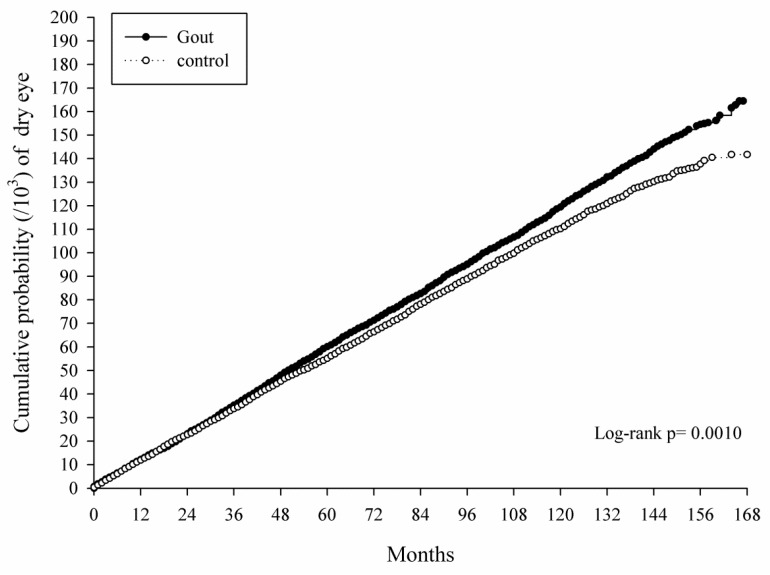
Kaplan–Meier curves with a cumulative proportion of dry eye disease in the study and matched control groups.

The Kaplan–Meier curves showed an increasing cumulative probability in the study group in an interval of up to 168 months, which was significantly higher than that in the control group (*p* = 0.0010) and was corrected to the length of disease course (Figure 2). Moreover, the Landmark analysis of aHR in the gout population with a different disease interval demonstrated that gout would lead to a significant risk of developing DED with a disease interval longer than 112 months while the risk of developing DED was numerically higher in the study group throughout the study period (Table 4).

## 4. Discussion

Briefly, the current study presented the increasing risk of DED in populations diagnosed with gout, evidenced by the higher crude HR and the significantly increased aHR after adjusting all the potential risk factors. Moreover, the cumulative probability of developing DED is also higher in patients with gout than the control group, which became significant 112 months after gout development. The other risk factors that elevate the occurrence of DED include older age, female gender, higher degree of urbanization, keratopathy, AMD, glaucoma, cataract, ischemic heart disease, hyperlipidemia, peripheral vascular disease, chronic pulmonary disease, rheumatic disease, peptic ulcer disease, liver disease, and malignancy.

In the current study, the aHR and cumulative probability results indicated that patients diagnosed with gout had a greater risk of DED than did non-gout individuals. In preceding studies, gout has already shown a significantly higher odds ratio of developing DED in regional population-based studies [17,18]. Our finding is consistent with previous experience with a significantly higher aHR. On the other hand, other previous studies indicated that gout is not an independent risk factor of DED [15,16,19]. However, all of those studies had limited case numbers and only adjusted the age and gender without considering the exposure interval of other potential risk factors while evaluating the relationship between gout and DED [15,16,17,18,19], which may be because the purpose of those studies mentioned above was to evaluate all possible risk factors of DED. In addition, the time sequence and disease interval of gout may also show the effect on the development of DED, in which the previous studies did not fully survey the possibility, since the exposure-to-outcome interval was absent [15,16,17,18,19]. To our knowledge, the current study was a preliminary investigation to explore the effect of gout on the occurrence of DED after adjusting multiple potential risk factors, considering the exposure period, which is positively correlated with the disease duration and became significant 112 months after gout occurrence. 

The occurrences of certain ocular diseases based on the co-morbidities included in the multivariate analysis were found to elevate the possibility of DED occurrence, which included keratopathy, glaucoma, cataract, and AMD. The exact mechanism result in the correlations between DED and these ocular diseases needs further investigation. Furthermore, several systemic diseases including rheumatic diseases, peptic ulcer disease, chronic pulmonary disease, liver disease, and some cardiovascular disorders showed a significant risk of developing DED. The significant effect of rheumatic disease (including rheumatic arthritis), chronic pulmonary diseases (including asthma), and hyperlipidemia at the onset of DED are correlated to the findings in previous studies conducted in different ethnicities [17,18]. Nevertheless, those systemic diseases may have minimal influence on the result since we enrolled all systemic diseases mentioned above into the multivariate analysis and gout is still an independent risk factor for DED. Thus, gout could be a risk factor of DED that is independent from rheumatic diseases, chronic pulmonary diseases, hyperlipidemia, and other components.

Although old age has been reported to be a risk factor for DED [25], there is no firm consensus on this conclusion [16,26]. In our study, the age ratio of the study group to the control group was 54.86 ± 17.02 years to 56.80 ± 16.76 years, and the number of patients aged older than 60 years was 13,554 and 15,305 in the study and control groups, respectively—the control group had a significantly older patient distribution (*p* < 0.0001). The multivariate analysis revealed that the effect of age on the risk of DED was significant in our study, with significantly higher aHR in the older population. Moreover, female sex may also alter the age-related influence on DED risk—this finding of the current study correlated to previous findings [19,27]. Furthermore, higher urbanization showed a significant risk of developing DED, suggesting that income level, which is usually higher in the urban area than the rural area, might play a role in the development of gout [28]. Regarding medications, anti-glaucoma agents have been shown to be associated with the development of DED [29,30]. Since glaucoma has been adjusted in the current study, the influence of such medications on the occurrence of DED may be minor. On the other hand, colchicine is commonly prescribed as a treatment for gout, while colchicine itself would lead to delayed corneal wound healing [31]. Still, the effect of colchicine on DED has not been fully elucidated—it warrants further evaluation.

Immunologically, the T-cell pathway associated with interleukin-6, interleukin-8, and tumor necrosis factor is enhanced in patients with gout, irrespective of acute or chronic status [11,21,32]. However, DED is considered as an inflammation-mediated disease, and experimental studies have demonstrated the activation of the T-cell pathway and elevated levels of interleukin and tumor necrosis factor in patients with DED compared with non-DED patients [33,34,35]. Clinically, meibomian gland dysfunction, a prominent risk factor of DED [36], is associated with hyperlipidemia [37]. Furthermore, hyperlipidemia status is also a prominent risk factor for gout [10], implying that a similar pathophysiology may exist in both gout and meibomian gland dysfunction, with the latter elevating the risk of DED. In addition, gout was found to be independently associated with DED after age and sex adjustment in previous studies [17,18], and while another study revealed a non-significant association between gout and DED, an odds ratio higher than one was still shown for these two diseases [16]. Consequently, DED among gout patients is not rare and an association could be possible, as supported in the current study.

The current study has some limitations. First, the observational and retrospective nature may restrict the accuracy and precision of the results. Second, because data on the uric acid level was missing, the severity of gout in each patient was unclear. In addition, the DED subtype was also unknown (i.e., evaporative-excess or meibomian gland dysfunction, and tear-deficiency subtype) since we only used the claimed data rather that real medical records. But since all DED subtypes are associated with an increment of inflammatory mediators, gout may have had a varying degree of effect on different subtypes of DED [4,38]. And although we enrolled the codes of laboratory tests to assume that the physician diagnosed gout after reading the positive laboratory report, this cannot be ensured, since the code can only present the arrangement of the laboratory test, while the examination results are not available.

Concerning the studies conducted by Farrand et al. and Dalbeth et al., 4% and 6.8% of adults in the US are diagnosed with gout and DED, respectively, which accounts for a large part of the population [3,10]. In terms of prevalence, nearly 9% of patients developed DED in the study group of the current study, which was significantly higher than the control group, and nearly two-folds numerically higher than the general prevalence of DED from the same population in a previous study [39], implying that DED is a common disorder in patients with gout, which is also a disease that influences a large proportion of patients. As a consequence, an ophthalmic examination may be suggested to patients with long-standing gout to disclose possible DED and treat promptly. Furthermore, the history of gout should be checked for patients with DED to determine it as a potential risk factor. However, whether the DED symptoms would improve after treating gout warrants further validation.

## 5. Conclusions

In conclusion, gout increases the risk of DED development after adjustment for other risk factors. Furthermore, the risk of DED increases with the duration of gout disease, which becomes significant after about 10 years of gout occurrence. Further large-scale prospective studies are warranted to investigate the effects of different forms of gout and different severities on the development of DED.

## Figures and Tables

**Table 1:** Characteristics in study groups at baseline between the study and matched control groups.

Variable	Gout *n* = 32,164	Control *n* = 32,164	*p* Value	ASD
Age at baseline, Mean ± SD	54.86 ± 17.02	56.80 ± 16.76	<0.0001	0.1148
<40	6502 (20.22%)	5493 (17.08%)		
40–59	12,108 (37.64%)	11,366 (35.34%)		
≥60	13,554 (42.14%)	15,305 (47.58%)		
Sex			0.1667	0.0806
Female	9839 (30.59%)	10,001 (31.09%)		
Male	22,325 (69.41%)	22,163 (68.91%)		
Urbanization			0.5120	0.0091
Urban	18,894 (58.74%)	18,960 (58.95%)		
Sub-urban	9750 (30.31%)	9775 (30.39%)		
Rural	3520 (10.94%)	3429 (10.66%)		
Low-income	229 (0.71%)	194 (0.60%)	0.0878	0.0135
Co-morbidities				
Keratopathy	1348 (4.19%)	1168 (3.63%)	0.0003	0.0289
AMD	320 (0.99%)	349 (1.09%)	0.2597	0.0089
Glaucoma	954 (2.97%)	855 (2.66%)	0.0182	0.0186
Cataract	4182 (13.00%)	4357 (13.55%)	0.0420	0.0160
Uveitis	165 (0.51%)	143 (0.44%)	0.2089	0.0099
Hypertension	15,837 (49.24%)	17,080 (53.10%)	<0.0001	0.0774
DM	8836 (27.47%)	9465 (29.43%)	<0.0001	0.0434
Ischemic heart diseases	2318 (7.21%)	2364 (7.35%)	0.4851	0.0055
Hyperlipidemia	13,302 (41.36%)	13,374 (41.58%)	0.5645	0.0045
Congestive heart failure	2772 (8.62%)	1903 (5.92%)	<0.0001	0.1042
Peripheral vascular disease	1503 (4.67%)	1127 (3.50%)	<0.0001	0.0591
Cerebrovascular disease	3917 (12.18%)	4223 (13.13%)	0.0003	0.0286
Dementia	619 (1.92%)	646 (2.01%)	0.4433	0.0061
Chronic pulmonary disease	7930 (24.65%)	6358 (19.77%)	<0.0001	0.1178
Rheumatic disease	1603 (4.98%)	439 (1.36%)	<0.0001	0.2075
Peptic ulcer disease	9066 (28.19%)	6132 (19.06%)	<0.0001	0.2160
Liver disease	11,990 (37.28%)	6177 (19.20%)	<0.0001	0.4098
Hemiplegia or paraplegia	621 (1.93%)	670 (2.08%)	0.1683	0.0109
Renal disease	4507 (14.01%)	1930 (6.00%)	<0.0001	0.2694
Malignancy	2060 (6.40%)	1551 (4.82%)	<0.0001	0.0688

ASD = absolute standardized difference, SD = standard deviation.

**Table 2:** Incidence risk of major events between the study group and matched control group.

Variable	Gout, *n* = 32,164	Control, *n* = 32,164
Dry eye		
Follow up person months	2,766,859	2,730,647
Event	2913	2631
Incidence rate * (95% CI)	105.28 (101.53–109.17)	96.35 (92.74–100.11)
Crude HR (95% CI)	1.093 (1.036–1.152)	Reference ^+^
aHR (95% CI)	1.065 (1.009–1.126)	Reference

Note: * per 100,000 person months, CI = confidential interval, aHR = adjusted hazard ratio which was adjusted for age, gender, urbanization, and both systemic as well as ocular co-morbidities. ^+^ The control group was applied as a standard to calculate the crude and adjusted hazard ratio of the study group based on the incidence rate between the two groups.

**Table 3:** The crude and adjusted hazard ratio of dry eye disease by univariate and multiple Cox regression model.

Variable	Crude HR (95% CI)	aHR (95% CI)
Exposure of gout (Ref: Non)		
Yes	1.093 (1.036–1.152)	1.065 (1.009–1.126)
Age at baseline (Ref: <40)		
40–59	2.828 (2.531–3.159)	2.204 (1.965–2.471)
≥60	4.687 (4.209–5.218)	2.886 (2.560–3.254)
Sex (Ref: Female)		
Male	0.436 (0.414–0.460)	0.564 (0.534–0.596)
Urbanization (Ref: Urban)		
Sub-urban	0.924 (0.871–0.981)	0.907 (0.855–0.963)
Rural	0.851 (0.776–0.933)	0.734 (0.669–0.806)
Co-morbidities		
Keratopathy	2.059 (1.851–2.291)	1.578 (1.416–1.758)
AMD	2.487 (2.048–3.020)	1.406 (1.154–1.713)
Glaucoma	2.547 (2.272–2.856)	1.531 (1.359–1.724)
Cataract	2.562 (2.409–2.726)	1.531 (1.427–1.643)
Uveitis	1.939 (1.441–2.609)	1.084 (0.801–1.467)
Hypertension	1.691 (1.603–1.784)	1.032 (0.972–1.097)
DM	1.472 (1.392–1.556)	0.991 (0.934–1.051)
Ischemic heart diseases	1.573 (1.440–1.718)	1.101 (1.005–1.206)
Hyperlipidemia	1.647 (1.562–1.736)	1.161 (1.097–1.228)
Congestive heart failure	1.358 (1.228–1.501)	0.868 (0.782–0.964)
Peripheral vascular disease	1.602 (1.427–1.797)	1.137 (1.012–1.279)
Cerebrovascular disease	1.350 (1.250–1.459)	0.949 (0.874–1.030)
Dementia	1.004 (0.784–1.284)	0.690 (0.538–0.887)
Chronic pulmonary disease	1.579 (1.490–1.673)	1.197 (1.127–1.272)
Rheumatic disease	1.693 (1.508–1.902)	1.331 (1.183–1.498)
Peptic ulcer disease	1.644 (1.555–1.739)	1.268 (1.196–1.345)
Liver disease	1.247 (1.180–1.319)	1.137 (1.072–1.205)
Hemiplegia or paraplegia	1.058 (0.868–1.291)	0.871 (0.711–1.067)
Renal disease	1.302 (1.197–1.417)	0.989 (0.907–1.079)
Malignancy	1.547 (1.390–1.721)	1.144 (1.027–1.274)

CI = confidential interval, Ref = reference, aHR = adjusted hazard ratio which was adjusted for age, gender, urbanization, and both systemic as well as ocular co-morbidities.

**Table 4:** Landmark analysis of the adjusted hazard ratio in different time intervals between the study and matched control groups.

Variable	0–56 Months	56–112 Months	112–168 Months
Incidence * in control	95.47 (90.65–100.55)	102.58 (96.22–109.36)	93.60 (83.55–104.85)
Incidence in gout	102.00 (97.02–107.22)	109.7 (103.17–116.64)	119.78 (108.5–132.22)
aHR (95% CI)	1.034 0.959–1.114)	1.057 (0.964–1.159)	1.270 (1.087–1.485)

Note: * Per 100,000 person months. CI = confidential interval, aHR = adjusted hazard ratio, which was adjusted for age, gender, urbanization, and both systemic as well as ocular co-morbidities.
